# Supplementary figures and images for: Effect of Integrin Blockade on Experimental Spondyloarthritis
Source: Biomolecules. 2024 Oct 31;14(11):1386. doi: 10.3390/biom14111386 (PMC11591768; doi:10.3390/biom14111386)

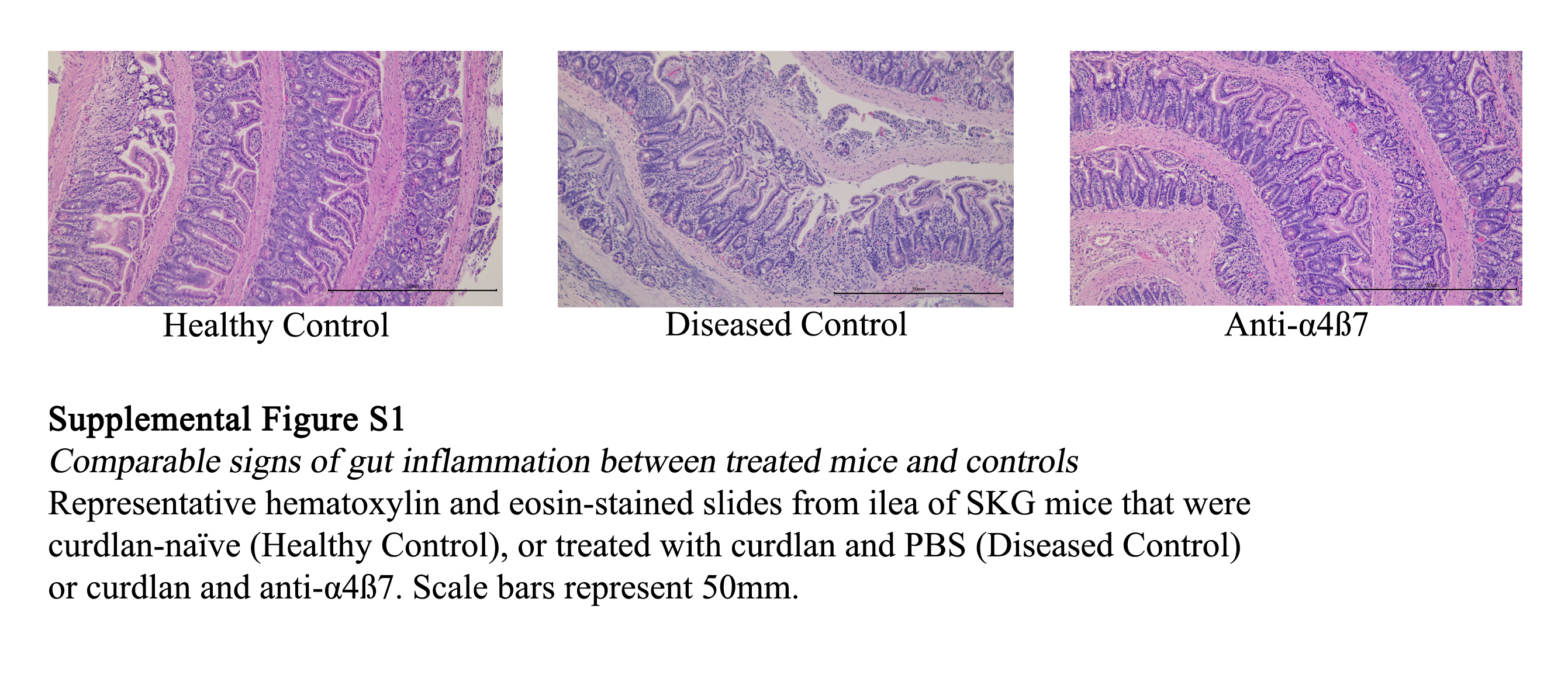

Supplement: Supplementary file 1 [file biomolecules-14-01386-s001.zip › Figure S1.png]

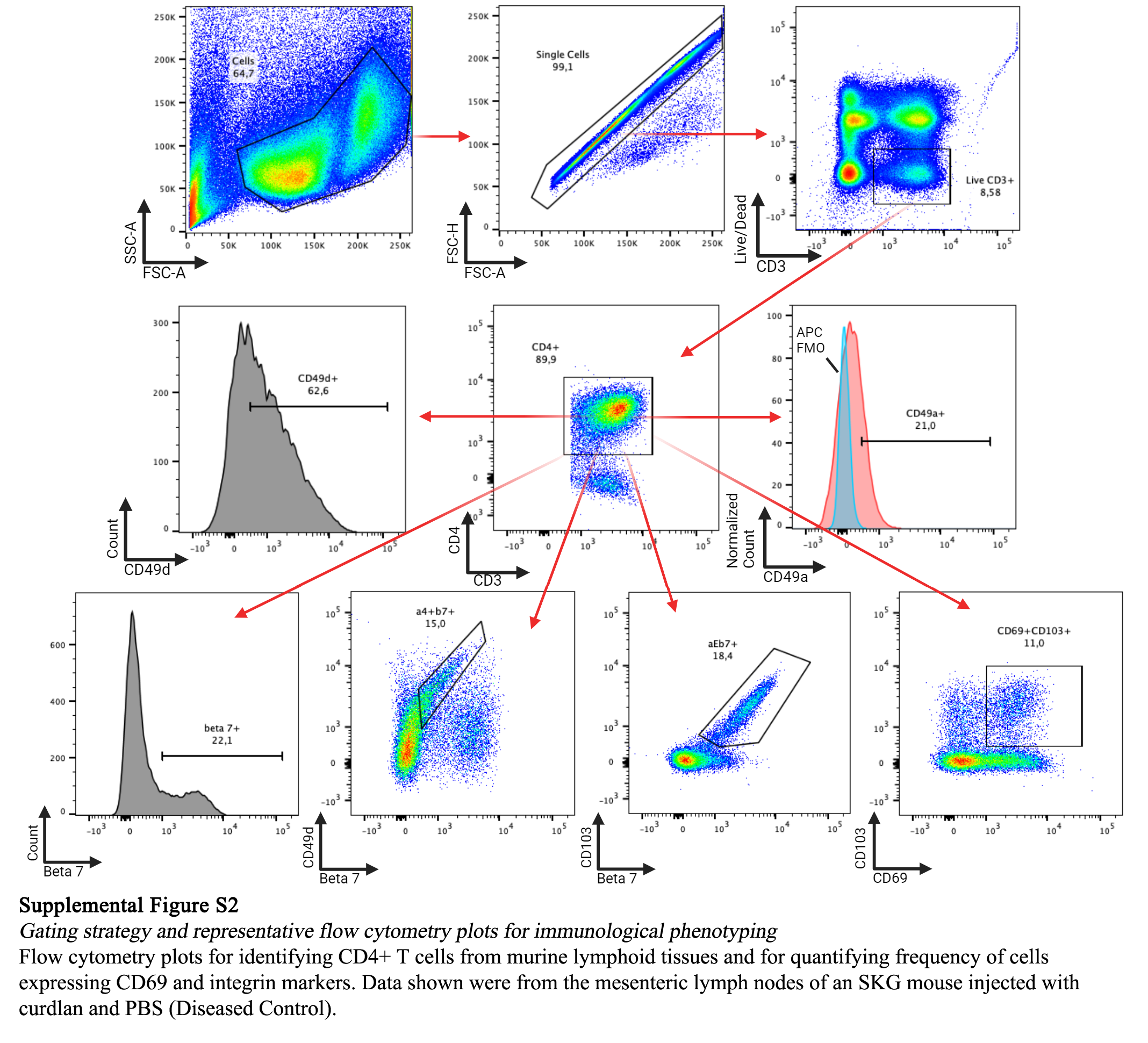

Supplement: Supplementary file 1 [file biomolecules-14-01386-s001.zip › Figure S2.png]
